# Myocardial Bridging-Induced Acute Coronary Syndrome: A Bridge Too Far

**DOI:** 10.7759/cureus.62052

**Published:** 2024-06-10

**Authors:** Priya Ramcharan, Arun R Katwaroo, Reyaz Hosein, Nicole Maharaj, Steven M Swarath, Valmiki Seecheran, Rajeev V Seecheran, Naveen A Seecheran

**Affiliations:** 1 Cardiology, North Central Regional Health Authority, Mt. Hope, TTO; 2 Internal Medicine, Trinidad Institute of Medical Technology, St. Augustine, TTO; 3 Internal Medicine, North Central Regional Health Authority, Mt. Hope, TTO; 4 Internal Medicine, University of Kansas Medical Center, Wichita, USA; 5 Cardiology, The University of the West Indies, St. Augustine, TTO

**Keywords:** widowmaker lesion, coronary artery disease (cad), atherosclerosis, acute coronary syndrome (acs), myocardial bridging (mb)

## Abstract

Recent studies suggest a potential association between myocardial bridging (MB) and accelerated atherosclerotic plaque formation. We describe the case report of a 37-year-old South Asian male with no established risk factors for coronary artery disease (CAD) who presented with a non-ST-segment-elevation acute coronary syndrome (NSTE-ACS) with a coincident widowmaker lesion and severe MB. He was successfully managed with comprehensive guideline-directed medical therapy (GDMT) and urgent percutaneous coronary intervention (PCI) of the culprit lesion, sparing the MB segment. The clinician should be cognizant of MB implicating ACS as a major adverse cardiovascular event (MACE) and its key management strategies.

## Introduction

Myocardial bridging (MB) is a congenital anomaly characterized by the segmental intramyocardial course of an epicardial coronary artery. This anomaly entails tunneling the affected artery segment beneath a muscular bridge formed by the overlying myocardium [1,2]. MB has been implicated in various clinical presentations, including exertional angina, acute coronary syndromes (ACS), lethal arrhythmias such as ventricular fibrillation (VF), stress-related cardiomyopathy (SRC), and even sudden cardiac death (SCD). However, these complications are infrequent, with the majority of MBs being asymptomatic and posing minimal clinical significance. Notably, previously asymptomatic individuals might develop symptoms due to the emergence of coexisting conditions, such as left ventricular hypertrophy (LVH), diastolic dysfunction, coronary vasospasm, or microvascular dysfunction. Furthermore, exercise and tachycardia can potentially exacerbate symptoms and trigger major adverse cardiovascular events (MACEs) [2].

Recent studies suggest a potential association between MB and accelerated atherosclerotic plaque formation. This phenomenon appears proximal to the bridged segment, with the tunneled portion seemingly spared from plaque accumulation. Endothelial injury within the proximal segment is postulated as a contributing factor, potentially triggered by increased local wall tension and stretch during the cardiac cycle [3]. This case report describes the potential association between MB and a widowmaker lesion (proximal left anterior descending artery (LAD) occlusion) precipitating a non-ST-segment-elevation ACS (NSTE-ACS) in a young South Asian male without any conventional cardiovascular risk factors.

## Case presentation

A 37-year-old Caribbean South Asian male with no significant medical history presented to the emergency department with an episode of typical angina, described as dull, substernal, and of 30 minutes duration. His family history revealed a first-degree relative (father, age 62) with coronary artery disease (CAD) status post percutaneous coronary intervention (PCI) for unstable angina. He is a lifelong non-tobacco user; however, he acknowledged occasional social alcohol consumption. He did not report any dyspnea, palpitations, presyncope, or syncope. He did not have any antecedent infection, nor did he have any pets or have traveled recently.

**Figure 1:**
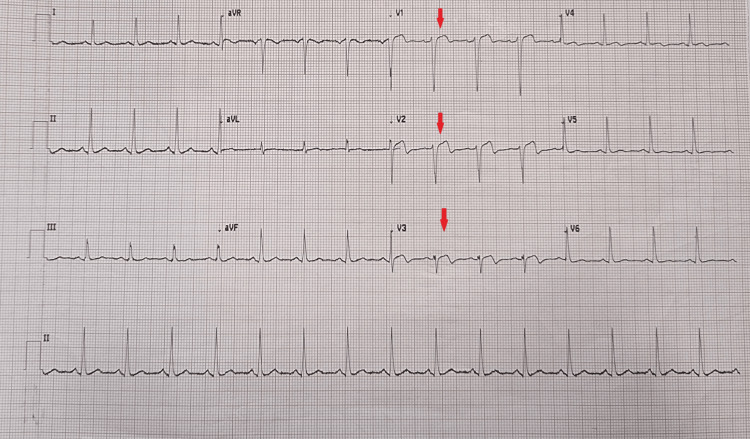
The patient’s 12-lead electrocardiogram. The downward red arrows illustrate the 2-millimeter ST-segment-elevation in leads V1-V3, suggestive of an evolving non-ST-segment-elevation acute coronary syndrome (NSTE-ACS) for which the cardiac catheterization laboratory was activated for urgent percutaneous coronary intervention (PCI).

**Figure 2:**
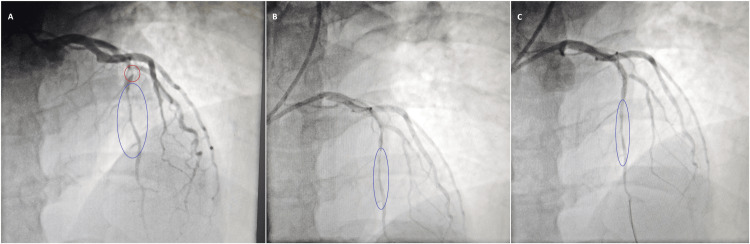
The patient’s coronary angiogram (CAG) and urgent percutaneous coronary intervention (PCI). (a) Left anterior oblique (LAO) imaging demonstrated the widowmaker lesion (encircled in red) and the severe mid-left anterior descending (LAD) artery myocardial bridging (MB) segment (ellipsed in blue). (b) LAO imaging demonstrated the pre-dilation of the culprit widowmaker lesion with a drug-coated balloon (DCB), with an improved but residual 50% stenosis and unchanged MB segment. (c) LAO imaging demonstration successful drug-eluting stent implantation at target lesion, sparing the MB segment.

Upon initial evaluation, the patient presented with vital signs, including blood pressure of 133/88 millimeters of mercury (mmHg), heart rate of 93 beats per minute (bpm), respiratory rate of 18 breaths per minute, oxygen saturation of 98% on 2 liters nasal cannula, and body temperature of 36.0 degrees Celsius (°C). Physical examination revealed no acute distress, with normal cardiopulmonary auscultation (regular heart sounds without murmurs and clear breath sounds bilateral). Furthermore, the neurologic and abdominal examinations were normal. The 12-lead electrocardiogram (ECG) demonstrated a sinus rhythm with 2 millimeters (mm) ST-segment-elevation in V1-V3, suggestive of an evolving non-ST-segment-elevation acute coronary syndrome (NSTE-ACS) (Figure 1). A high-sensitivity cardiac troponin I (hs-cTnI) returned as 8.8 ng/dL (normal 0.0-0.8 ng/dL) (Table 1). The patient proceeded directly to the cardiac catheterization laboratory for urgent PCI. During coronary angiography (CAG), there was a “widowmaker” lesion (proximal LAD occlusion) with severe mid-LAD intra-MB, near-complete systolic compression, and a “milking” phenomenon [4]. The culprit lesion was successfully treated with provisional stenting, comprising pre-dilation with a drug-coated balloon (DCB) and subsequently stented with a zotarolimus drug-eluting (ZES) stent, with care to exclude the bridging segment with a good angiographic result and no complications (Figures 2a-2c). No intravascular ultrasonography (IVUS), fractional flow reserve (FFR), or optical coherence tomography (OCT) was performed, as these were not currently available in our setting.

**Table 1:** The patient’s initial diagnostic data.

Diagnostic data			
Complete Blood Count	Patient value	Units	Normal range
White blood cells (WBC)	6.4	10^3^µL	4.0 – 10.3
Red blood cells (RBC)	4.72	10^6^µL	4.38 – 5.77
Hemoglobin (Hb)	16.1	g/dL	13.6 – 17.2
Hematocrit (HCT)	48.30	%	39.5 – 50.30
Mean corpuscular volume (MCV)	86.1	fL	80.70 – 95.50
Mean corpuscular hemoglobin (MCH)	31.7	pg	27.2 – 33.50
Mean corpuscular hemoglobin concentration (MCHC)	32.9	g/dL	32.7 – 35.6
Red cell distribution width (RCW)	12.8	%	11.8 – 14.3
Platelet (PLT)	310	10^3^µL	159 – 388
Mean platelet volume (MPV)	9.2	fL	6.90 – 10.8
Comprehensive metabolic panel	Patient value	Units	Normal range
Sodium (Na)	140.0	mmol/L	135 – 145
Potassium (K)	4.4	mmol/L	3.5 – 5.1
Creatinine (Cr)	0.8	mg/dL	0.7 – 1.2
Urea (BUN)	193.0	mg/dL	6.0 – 23.0
Calcium (Ca)	9.1	mg/dL	8.6 – 10.2
Aspartate transferase (AST)	32	IU/L	5.0 – 40.0
Alanine transaminase (ALT)	28	IU/L	5.0 – 41.0
Alkaline Phosphatase (ALP)	110	U/L	40 – 129
Gamma-glutamyl transferase (GGT)	34	U/L	6.0 – 61.0
Total bilirubin (TBili)	0.48	mg/dL	0.0 – 1.29
Direct bilirubin (DBili)	0.25	mg/dL	0.0 – 0.4
Indirect bilirubin (IBili)	0.23	mg/dL	0.0 – 1.0
Total protein (TP)	8.1	g/dL	6.4 – 8.3
Albumin (ALB)	4.8	g/dL	3.5 – 5.2
Globulin (GLB)	33.4	g/dL	2.3 – 4.5
Magnesium (Mg)	1.8	mg/dL	1.6 – 2.4
Phosphorus (Phos)	3.5	mg/dL	2.5 – 4.5
Cardiac Pack	Patient value	Units	Normal range
C-reactive protein (CRP)	20.10	mg/dL	0.100 – 0.500
High-sensitivity cardiac Troponin I (hs-cTnI) 1	8.8	ng/dL	0.0 – 0.8
High-sensitivity cardiac Troponin I (hs-cTnI) 2	3.7	ng/dL	0.0 – 0.8
D-dimer	0.26	ng/mL	< 0.5
B-type natriuretic peptide (BNP)	112	pg/mL	< 100
Total cholesterol	174	mg/dL	< 200
Low-density lipoprotein (LDL)	136	mg/dL	60 - 130
High-density lipoprotein (HDL)	38	mg/dL	> 40
Triglycerides (TG)	108	mg/dL	< 150
Glycosylated hemoglobin (HbA1c)	5.4	%	< 6.5%

The patient was hospitalized post-PCI for 48 hours. He received comprehensive, guideline-directed medical therapy (GDMT), which comprised aspirin, ticagrelor, ramipril, carvedilol, eplerenone, empagliflozin, rosuvastatin, amlodipine, and colchicine in accordance with American College of Cardiology and American Heart Association standard-of-care practice. A chest radiograph (CXR) reported no cardiomegaly or pulmonary edema. A two-dimensional transthoracic echocardiogram (2D-TTE) demonstrated mild anterior hypokinesis with an estimated left ventricular ejection fraction (LVEF) of 45%-50%. The remainder of his ensuing hospitalization was uneventful as he remained angina-free and hemodynamically stable with down-trending cardiac biomarkers. He was discharged with a follow-up appointment at the cardiology clinic in one week.

## Discussion

MB is a medical condition first observed by Reyman in 1737 during an autopsy and was later described in 1960 through angiography [5-7]. This condition occurs when a part of the coronary artery runs through the myocardium instead of epicardially, creating a tunnel-like pathway [7,8]. Studies have shown that MB may be highly underdiagnosed, with postmortem examination reports indicating an incidence rate ranging from 15% to 85%, with a mean of 25% [5,7]. Cardiac computer tomography angiography (CCTA) follows closely at 22%, while invasive CAG displays a rate of 1.5%-16% (without provocation), with the majority being discovered incidentally [5,9]. Male patients are affected in 70% of cases [10]. The disparity in diagnostic studies can be attributed to including non-functional myocardial loops in pathological studies, advanced imaging, and hemodynamic modalities such as IVUS, FFR, OCT, and the lack of provocative testing [6,7,10]. MB was historically considered a benign anomaly due to the predominance of diastolic coronary perfusion. Although it is commonly believed to be low-risk, MBs can have severe clinical consequences such as angina, lethal arrhythmias, heart failure (HF), and SCD [6,7]. Accurate diagnosis and management of patients displaying symptomatology are integral to identifying potential risks and mitigating MACE.

However, recent evidence suggests a more complex relationship between anatomical factors and their pathophysiology. A broad spectrum of clinical presentations is contingent on the tunneled segment’s location, length, and depth, which underpin the potential for ischemia and guiding treatment decisions [11]. The LAD is the most common site in 67%-98% of detected myocardial bridges and most at the mid-LAD segment [10,11]. The length varies from 4 to 40mm, and depth ranges from 10 to 30mm [6,7,9,12,13]. Treatment strategies, especially surgical interventions, are also affected by this depth. Additionally, the length of the tunneled segment is pivotal as it can impact the number of branches affected by MB, particularly in the context of LAD MBs and their potential to affect diagonal or septal branches. This aspect holds significant clinical relevance [11]. Based on current classifications, it appears that individuals with isolated MB, which refers to MB without any accompanying CAD, do not demonstrate an increased incidence of high-risk stress test findings, which is diametrically opposite to the concept that MB inevitably results in impaired coronary blood flow (CBF). The intracoronary hemodynamic assessment estimates the functional significance of MB via pressure gradients and can provide valuable insights into inducible ischemia [14].

Almost 70%-95% of CBF in the coronary arteries occurs during the diastole when the myocardium relaxes, and there is no arterial compression [9]. Although an MB during systole theoretically should not affect CBF, studies revealed that arterial compression caused by a myocardial contraction can affect localized vasospasm [5,11]. As a result, there is a reduction of the diameter of the coronary artery by 34%-51%, an increase in blood flow velocities and retrograde flow, and reduced coronary flow reserve (CFR) - particularly in times of stress and exercise. The increased sympathetic tone from these states can induce tachycardia, resulting in decreased diastolic perfusion time and compromised myocardial perfusion [10]. Paradoxically, the vessel segment proximal to the MB demonstrates a significantly increased susceptibility to atherosclerosis, with reported rates approaching 90% attributed to hemodynamic alterations [7]. This accelerated plaque formation may be linked to changes in shear stress, which shifts from low shear stress at the tunnel entrance to high shear within the tunnel [7]. Endothelial cell morphology analysis at the tunneled segment entrance reveals a unique structure described as “flat, polygonal, and polymorphic,” indicating a low shear stress state. In contrast, within the MB, they exhibit a helical orientation, typically associated with laminar flow and high shear stress. The alteration in shear stress, the frictional force to flow, is proatherogenic and pro-inflammatory, leading to endothelial dysfunction and atherosclerosis development [15]. Additionally, there is an intricate milieu of vasoactive compounds such as endothelial nitric oxide synthase (eNOS), endothelin-1, and angiotensin-converting enzyme (ACE) in the proximal segment, which impact smooth muscle cell proliferation and atheroma formation. The presence of cardiovascular risk factors such as hypertension (HTN), diabetes mellitus (DM), and dyslipidemia (HLD) has been shown to amplify CAD proximal to the MB [15]. The MB segment is usually spared from atherosclerotic disease due to a thinner intima absence of smooth muscle cells and foam cells, which are integral in atherogenesis [7,10].

Electrocardiographic changes are frequent, but there are reported cases of pseudo-Wellens’ (biphasic T-waves or deep T-wave inversions in leads V2 and V3), VF, transient STE, and atrioventricular block [9,11,16,17]. Echocardiographic findings have revealed an abnormal left ventricular strain pattern, diastolic dysfunction, myocardial stunning, and SRC. Additionally, MB has been linked to LVH and hypertrophic cardiomyopathy (HCM) as it accentuates the supply-demand mismatch and augments compression of the microvascular vasculature [16,18]. Stress echocardiography may uncover septal bulking (a transient focal septal wall motion with apical sparing) and an abnormal strain pattern [9,19]. The classic angiographic feature is the systolic narrowing of the vessel, commonly called “milking,” which is characteristically described as a “step-down” to “step-up” phenomenon. This occurs when there is a greater than 70% reduction in the systolic luminal diameter, with complete or partial reduction in the luminal diameter persisting during systole. Despite this description, this is only demonstrated in 5% of cases [11].

Currently, management strategies primarily focus on pharmacotherapies such as beta-blockers (βBs) and non-dihydropyridine calcium-channel blockers (CCBs) [7]. Mitigating the hemodynamic consequences and reducing compression of the bridged segment are the mainstays of treatment. Protracting diastole with βBs could enhance CBF by extending the perfusion period. Additionally, reduced heart rates attenuate myocardial contractility, resulting in the lessening of systolic compression on the MB. CCBs offer a potential benefit in cases with suspected vasospasm due to their vasodilatory properties [11]. However, nitrates are generally contraindicated due to their ability to increase systolic compression, enhancing vessel wall compliance and contractility, potentially exacerbating the compression. As there is an emerging link between MB and accelerated atherosclerosis, the use of antiplatelets and statins have also been utilized [20]. In severe, refractory cases with persistent symptoms, surgical interventions like myotomy (resection of overlying muscle fibers), intracoronary stenting, and coronary artery bypass grafting (CABG) may be considered, however, long-term efficacy remains uncertain and requires further investigation [7]. There have been reports of stent fracture, in-stent restenosis (ISR), perforation, coronary artery dissection, and risk of graft occlusion [10].

Our patient displayed several key facets of MB, such as being male gender, the MB site being proximal to mid-LAD, approximately 30 mm in length. The compelling link was the presence of the widowmaker lesion despite his young age and the absence of conventional risk factors such as HTN, DM, HLD, and tobacco use. It must be acknowledged that the patient is of South Asian descent, and there were no lipoprotein(a) levels available in our setting, both of which are linked to increased, premature MACE [21-23]. Additionally, a dedicated CCTA was not performed. Thus, while we cannot definitively exclude that the widowmaker lesion was solely attributed to the severe MB, based on the above, the clinical scenario did not fit the classic de novo plaque rupture picture.

## Conclusions

We describe the case report of a 37-year-old South Asian male with no established risk factors for CAD who presented with an NSTE-ACS with a coincident widowmaker lesion and severe MB. He was successfully managed with comprehensive GDMT and urgent PCI of the culprit lesion, sparing the MB segment. The definitive role of MB in atherosclerotic plaque formation within the Caribbean population warrants further investigation.

## References

[1] Angelini P, Velasco JA, Flamm S (2002). Coronary anomalies: incidence, pathophysiology, and clinical relevance. Circulation.

[2] Corban MT, Hung OY, Eshtehardi P (2014). Myocardial bridging: contemporary understanding of pathophysiology with implications for diagnostic and therapeutic strategies. J Am Coll Cardiol.

[3] (2024). Myocardial bridging in adults. https://www.acc.org/latest-in-cardiology/articles/2020/08/04/08/48/myocardial-bridging-in-adults.

[4] Holmes DR Jr, Bell MR (2000). Left anterior descending artery stenosis: the widow maker revisited. Mayo Clin Proc.

[5] Thej MJ, Kalyani R, Kiran J (2012). Atherosclerosis and myocardial bridging: not a benign combination. An autopsy case report. J Cardiovasc Dis Res.

[6] Chatzizisis YS, Giannoglou GD (2009). Myocardial bridges spared from atherosclerosis: overview of the underlying mechanisms. Can J Cardiol.

[7] Lee MS, Chen C-H (2015). Myocardial bridging: an up-to-date review. J Invasive Cardiol.

[8] Freiling TP, Dhawan R, Balkhy HH, Castillo J, Cotter EK, Chaney MA (2022). Myocardial bridge: diagnosis, treatment, and challenges. J Cardiothorac Vasc Anesth.

[9] Ki YJ (2021). Myocardial bridging presenting as myocardial ischaemia induced cardiac arrest: a case report. BMC Cardiovasc Disord.

[10] Matta A, Canitrot R, Nader V (2021). Left anterior descending myocardial bridge: angiographic prevalence and its association to atherosclerosis. Indian Heart J.

[11] Sternheim D, Power DA, Samtani R, Kini A, Fuster V, Sharma S (2021). Myocardial bridging: Diagnosis, functional assessment, and management: JACC state-of-the-art review. J Am Coll Cardiol.

[12] Tanitame N, Tanitame K (2023). Deep and long myocardial bridge. QJM.

[13] Gould KL, Johnson NP (2015). Myocardial bridges: lessons in clinical coronary pathophysiology. JACC Cardiovasc Imaging.

[14] Schwarz ER, Gupta R, Haager PK, vom Dahl J, Klues HG, Minartz J, Uretsky BF (2009). Myocardial bridging in absence of coronary artery disease: proposal of a new classification based on clinical-angiographic data and long-term follow-up. Cardiology.

[15] Villela PB, Oliveira GM (2019). A new marker of myocardial bridge?. Arq Bras Cardiol.

[16] Milne D, Ramadhin D, Seecheran R, Seecheran V, Henry R, Seecheran NA (2022). The curious case of pseudo-Wellens’ syndrome and myocardial bridging. J Investig Med High Impact Case Rep.

[17] Lin CC, Lai CH, Lin WS, Lin CS (2021). Severe myocardial bridge presenting as paroxysmal atrioventricular block. J Postgrad Med.

[18] Zerbo S, Lanzarone A, Raimondi M, Martino L, Malta G, Cappello F, Argo A (2020). Myocardial bridge pathology and preventable accidents during physical activity of healthy subjects: a case report and a literature review. Med Leg J.

[19] Jhi J-H, Cho K-I, Ha J-K (2011). Alteration of left ventricular function with dobutamine challenge in patients with myocardial bridge. Korean J Intern Med.

[20] Patel M, Swofford B, Distler E (2017). Myocardial bridge: Bridging the differential diagnosis. BMJ Case Rep.

[21] Patel AP, Wang M, Kartoun U, Ng K, Khera AV (2021). Quantifying and understanding the higher risk of atherosclerotic cardiovascular disease among South Asian individuals: results from the UK Biobank prospective cohort study. Circulation.

[22] Volgman AS, Palaniappan LS, Aggarwal NT (2018). Atherosclerotic cardiovascular disease in South Asians in the United States: epidemiology, risk factors, and treatments: a scientific statement from the American Heart Association. Circulation.

[23] Miksenas H, Januzzi JL Jr, Natarajan P (2021). Lipoprotein(a) and cardiovascular diseases. JAMA.

